# Efficacy of Adeno-Associated Virus Serotype 9-Mediated Gene Therapy for AB-Variant GM2 Gangliosidosis

**DOI:** 10.3390/ijms241914611

**Published:** 2023-09-27

**Authors:** Meera Vyas, Natalie M. Deschenes, Karlaina J. L. Osmon, Zhilin Chen, Imtiaz Ahmad, Shalini Kot, Patrick Thompson, Chris Richmond, Steven J. Gray, Jagdeep S. Walia

**Affiliations:** 1 Centre for Neuroscience Studies, Queen’s University, Kingston, ON K7L 3N6, Canada; 2 Department of Biomedical and Molecular Sciences, Queen’s University, Kingston, ON K7L 3N6, Canada; zc@queensu.ca (Z.C.); 3 Department of Pediatrics, Queen’s University, Kingston, ON K7L 2V7, Canada; pt24@queensu.ca; 4 Department of Pediatrics, UT Southwestern Medical Center, Dallas, TX 75390, USA

**Keywords:** GM2 gangliosidosis, gene therapy, adeno-associated virus, GM2 ganglioside, GM2 activator protein, AAV9, intravenous

## Abstract

GM2 gangliosidoses are a group of neurodegenerative lysosomal storage disorders that are characterized by the accumulation of GM2 gangliosides (GM2), leading to rapid neurological decline and death. The hydrolysis of GM2 requires the specific synthesis, processing, and combination of products of three genes—*HEXA*, *HEXB*, and *GM2A*—within the cell’s lysosomes. Mutations in these genes result in Tay-Sachs disease, Sandhoff disease, or AB-variant GM2 gangliosidosis (ABGM2), respectively. ABGM2, the rarest of the three types, is characterized by a mutation in the *GM2A* gene, which encodes the GM2 activator (GM2A) protein. Being a monogenic disease, gene therapy is a plausible and likely effective method of treatment for ABGM2. This study aimed at assessing the effects of administering a one-time intravenous treatment of single-stranded Adeno-associated virus serotype 9 (ssAAV9)-*GM2A* viral vector at a dose of 1 × 10^14^ vector genomes (vg) per kilogram per mouse in an ABGM2 mouse model (*Gm2a*^−/−^). *ssAAV9-GM2A* was administered at 1-day (neonatal) or 6-weeks of age (adult-stage). The results demonstrated that, in comparison to *Gm2a*^−/−^ mice that received a vehicle injection, the treated mice had reduced GM2 accumulation within the central nervous system and had long-term persistence of vector genomes in the brain and liver. This proof-of-concept study is a step forward towards the development of a clinically therapeutic approach for the treatment of patients with ABGM2.

## 1. Introduction

GM2 gangliosidoses are a set of autosomal recessive lysosomal storage disorders that result from the chronic accumulation of GM2 ganglioside (GM2) within the central nervous system (CNS). Cytotoxic buildup of GM2 results in extensive neuronal apoptosis, ultimately causing neurodevelopmental delays, regression of motor milestones, and premature death [1]. The three known types of GM2 gangliosidosis are Tay-Sachs Disease (TSD), Sandhoff Disease (SD), and AB-variant GM2 gangliosidosis (ABGM2), which manifest through a partial or complete loss-of-function mutation in genes encoding β-N-acetylhexosaminidase A (β-HexA) or GM2 activator (GM2A) proteins, both required for GM2 catabolism.

β-HexA is a heterodimeric hydrolase composed of two subunits (α- and β-subunits), encoded by the *HEXA* (TSD) and *HEXB* (SD) genes, respectively. The α- and β-subunits can also dimerize to form HexS (αα) and HexB (ββ), isozymes of β-HexA; however, β-HexA is the only one of these enzymes capable of hydrolyzing GM2. Proper catabolism also requires the activity of the GM2A protein, which binds and removes GM2 from lysosomal membranes and presents it to β-HexA [2,3,4]. Destabilizing mutations in *GM2A*, which encodes the GM2A protein, lead to ABGM2, the rarest and likely most underreported form of GM2 gangliosidosis. There are less than 30 reported cases and a likely incidence of <1 in every million live births [1,5,6]. As a comparison, the incidence for TSD is reported to be approximately 1 in 320,000 and for SD to be approximately 1:130,000, with incidence being higher in some populations [7,8,9,10]. The β-HexA enzyme functions at normal levels in patients with ABGM2, although the reduced or absent expression of GM2A protein results in impaired breakdown of GM2 by β-HexA [11].

The clinical manifestation of GM2 gangliosidosis is dependent upon the residual protein activity, with the infantile form being the most prevalent and severe. Juvenile and adult forms can be relatively mild; however, quality of life is compromised in later stages of the disease [5,12]. Infants suffering from GM2 gangliosidosis show normal development during the early stages of infancy, up to 4–6 months of age, followed by a delay in development and eventual regression and loss of previously attained milestones [12]. Symptoms include progressive neuromuscular degeneration and weakness, an exaggerated startle response, loss of vision accompanied by a cherry-red spot in the retina’s fundus, dysphagia, seizure activity and macrocephaly [1,5,6]. At present, curative interventions for GM2 gangliosidoses remain elusive, and the primary focus of clinical management is palliative care.

Pharmacological chaperones [13,14], enzyme replacement therapies [15,16,17], substrate reduction therapies [18,19,20,21,22], and stem cell therapies [23] have all previously been trialed with some success in pre-clinical and/or clinical trials. However, obstacles to some of these treatment modalities remain. For example, frequent and repeated administration [24], inability to cross the blood-brain barrier (BBB) [25], graft-versus-host disease [26] and efficacy that is limited to patients with specific mutations [27,28]. A promising treatment option for these diseases is gene therapy, which introduces a functional gene into the cells of the affected individual in hopes of restoring depleted protein levels [28]. Notably, there has been a surge in gene therapy investigations for TSD and SD [29,30,31,32,33,34,35,36], with currently active and ongoing clinical trials (NCT04798235 and NCT04669535). However, research endeavors pertaining to ABGM2 have yet to gain commensurate momentum and require further attention.

Rapid progress in viral technology has accelerated the development of gene therapies for neurological disorders in a clinical setting [37]. The choice of an appropriate vector for transgene delivery is crucial for achieving maximal transduction and optimizing treatment efficacy. Recombinant adeno-associated viruses (rAAV) have emerged as a preferred vector in gene therapy due to their numerous advantages. Compared to other vectors, such as retroviruses, rAAVs have been shown to be non-pathogenic so far and elicit fewer adverse events, like oncogene integration [38]. Furthermore, they can transduce both dividing and non-dividing cells [39,40]. Among the naturally occurring serotypes, the AAV serotype (AAV9) stands out as the most promising for CNS-based therapies. AAV9 can traverse the BBB through the transcytosis of endothelial cells [41,42] and has axonal transport competence [43], which allows for extensive neuronal transduction throughout the CNS.

This study assesses the potential of *ssAAV9-GM2A*, which carries a functional human *GM2A* transgene, to biochemically rescue GM2 breakdown in a mouse model deficient in GM2A protein (*Gm2a*^−/−^). At twenty weeks of age, these mice display moderate accumulation of GM2 in the brain, leading to behavioral impairments by 27 weeks of age. While this model presents with some neurological and behavioral setbacks, their life expectancy remains normal, with a lifespan of up to 104 weeks of age [44]. This intermediate phenotype stands in contrast to the TSD (*Hexa*^−/−^) and SD (*Hexb*^−/−^) murine models, with TSD mice exhibiting no behavioral or biochemical impairments and a normal lifespan, while SD mice experience cytotoxic GM2 accumulation, motor impairment, and early death at 14–16 weeks of age [45]. Phenotypically and biochemically, *Gm2a*^−/−^ mice closely resemble the human adult-onset form of ABGM2, whereas SD mice are more comparable to the juvenile onset of the disease [1].

**Figure 1:**
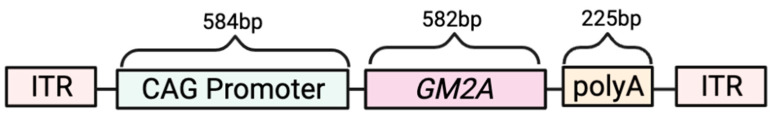
*ssAAV9*-*GM2A* construct design. Inverted terminal repeats (ITRs) flank the promoter, transgene, and polyadenylation sequence (polyA). The CAG promoter was synthesized by fusing the CMV enhancer with a chicken β-actin (CBA) transcription start site and a rabbit β-globin intron. GM2A: *GM2A* activator gene. polyA: polyadenelation sequences. bp: base pairs.

This proof-of-concept study evaluated the efficacy of systemically administered *ssAAV9*-*GM2A* (Figure 1) in an ABGM2 mouse model during the neonatal stage (1-day old) or adult stage (6 weeks of age). A dose of 1.0 × 10^11^ vector genomes (vg) per mouse was utilized in this study, as it is consistent with those observed in present human clinical trials [46]. The safety, stability, and effectiveness of *ssAAV9*-*GM2A* were evaluated by comparing early treatment responses (14 weeks post-injection) to those observed over a more extended period of up to 1-year post-injection. Our findings suggest that *ssAAV9*-*GM2A* is a safe and well-tolerated therapeutic approach capable of ameliorating the biochemical accumulation of GM2 in the CNS while persisting stably in the long term (1-year post-injection). The results of this investigation represent a promising advancement towards the development of a clinically efficacious treatment strategy for patients with ABGM2. Moreover, our study contributes to the growing body of knowledge surrounding ABGM2, thus attempting to bridge critical gaps in this field. 

## 2. Results

### 2.1. Biodistribution of ssAAV9-GM2A in the Brain and Liver of Mice 14-Weeks and 1-Year Post-Injection

**Figure 2:**
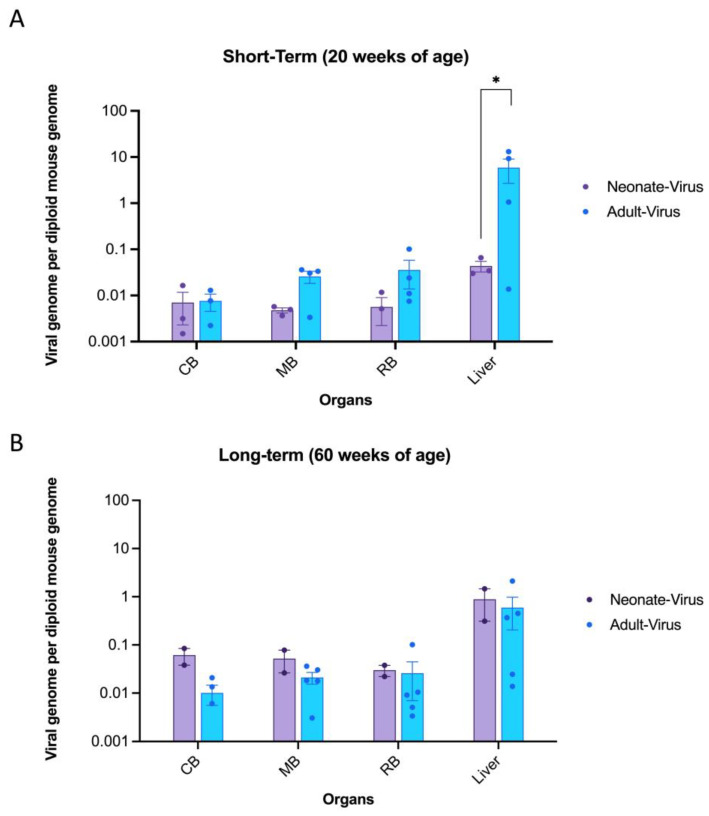
*GM2A* transgene distribution in the liver and three parts of the brain following intravenous injection of *ssAAV9-GM2A* in neonatal (1 day old) and adult-injected mice (6 weeks of age). *GM2A* transgene was not detectable in *Gm2a*^−/−^ mice that were dosed with vehicle and GFP virus; therefore, those cohorts are not shown on the graph. The data are presented as the copies of vector DNA per diploid mouse genome (*LaminB2*) found in each assessed organ. Data are expressed as mean ± SEM. CB: caudal section of the brain. MB: mid-section of the brain. RB: rostral section of the brain. (**A**) *GM2A* copy number in short-term cohorts. The transgene is robustly expressed in the liver and throughout the brain at 20 weeks of age (*n* = 3 or 4/cohort; 1-way ANOVA). Notably, the biodistribution in the liver was significantly higher in the adult-treated mice versus the neonate-treated mice (*p* < 0.0286 [*]; *n* = 3 or 4/cohort; 1-way ANOVA). (**B**) *GM2A* copy number in long-term cohorts. The transgene is detectable in the liver and throughout the brain 1-year post-injection (60 weeks of age; *n* = 2 or 5/cohort; 1-way ANOVA). There were no significant differences seen between the neonate (*n* = 2/cohort) and adult-treated (*n* = 5/cohort) in any of the analyzed organs (1-way ANOVA).

In this study, a quantitative polymerase chain reaction (qPCR) assay was used to analyze the biodistribution of the transgene (*GM2A*) in the brain and liver samples of each mouse. Biodistribution in the liver and brain samples was determined based on the diploid mouse genome (Figure 2). These findings indicated that the *GM2A* transgene was retained in all three brain regions in both neonatal- and adult-treated mice for short-term (Figure 2A) and long-term (Figure 2B) cohorts, demonstrating long-term retention of the transgene. While the short-term adult-treated cohort appeared to have more transduced cells than the neonatally treated cohort, the opposite was observed in the long-term cohorts for both the brain and liver samples. Notably, biodistribution in the CNS increased over time, with the long-term cohorts displaying a larger copy number than the short-term cohorts. This may be attributed to human error during intravenous injections or to the possibility that some transduced cells migrated to the brain and contributed to the observed copy number.

Moreover, the liver had a significantly higher transgene presence than the neonate-injected mice (Figure 2A), as the liver is a target organ for AAV, which is evident in the biodistribution data presented. However, this disparity in copy number between the brain and liver appeared to equalize in the long-term mice (Figure 2B). Overall, these data suggested that regardless of the age of the mice, *GM2A* transgene persisted in all organs of the CNS and liver up to 1-year post-injection.

### 2.2. ssAAV9-GM2A Drives GM2A Protein Expression In Vitro and In Vivo

**Figure 3:**
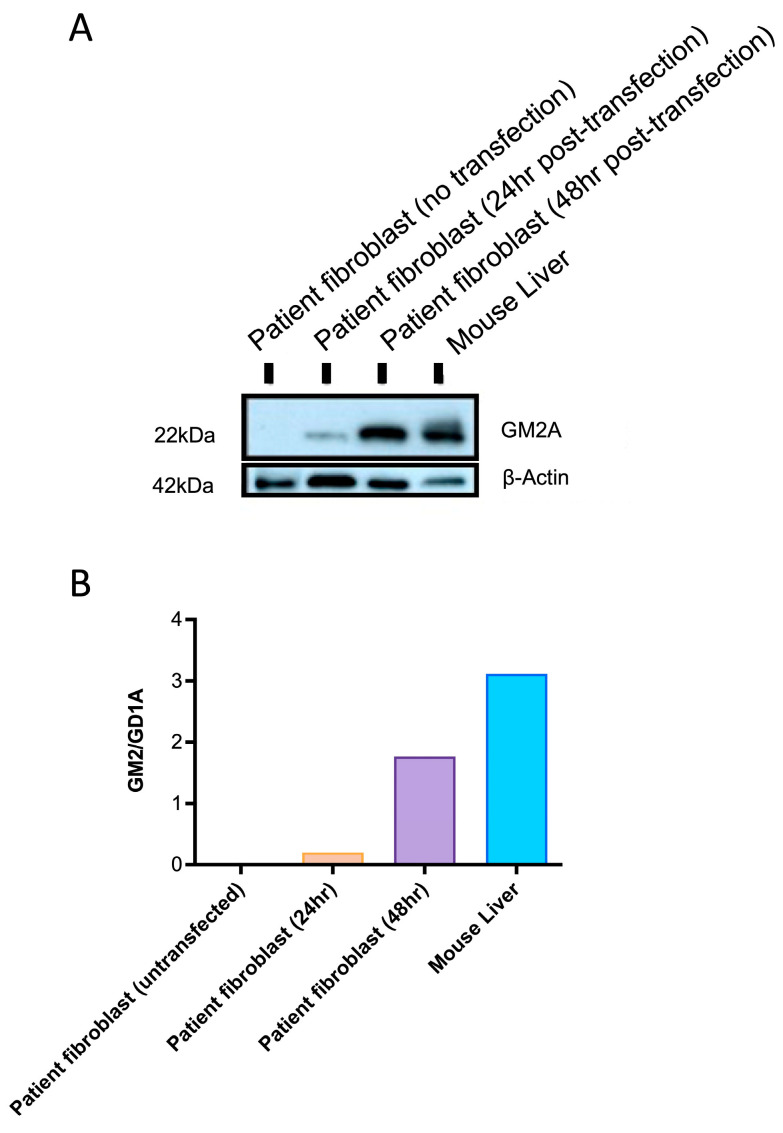
(**A**) A Western blot was conducted for confirmatory GM2A protein expression mediated by a plasmid harboring the *GM2A* transgene, or *ssAAV9-GM2A* (in vitro and in vivo). Fibroblasts from patients with ABGM2 who have a loss-of-function mutation in *GM2A* (*GM2A*^−/−^) were transfected with a plasmid containing *GM2A* and collected 24- or 48 h post-transfection. This can be seen in the second and third columns, respectively. The fourth column displays Western blot results from liver tissue that was collected from a *Gm2a*^−/−^ mouse treated with ssAAV9-*GM2A* at 6-weeks of age. The liver was collected at 20 weeks of age, 14-weeks post-injection. β-actin protein was used as a housekeeping protein (42 kDA). (**B**) Quantification of the GM2AP signal from the western blots: The intensity of the GM2A protein (~22 kDA) was quantified by densitometry and normalized to β-actin intensity.

Western blot analysis was done using fibroblasts from patients with ABGM2 and liver cells from *Gm2a*^−/−^ mice treated with *ssAAV9-GM2A* to confirm GM2A protein expression. Patient fibroblasts were cultured and transfected with a plasmid harboring *GM2A* and collected 24- or 48-h post-transfection. Patient fibroblasts that were not transfected were used as a negative control. Additionally, liver cells were collected from a *Gm2a*^−/−^ mouse that was treated with *ssAAV9-GM2A* through an intravenous injection at 6-weeks of age and euthanized at 20-weeks of age (14-weeks post-injection). As expected, GM2A protein expression was not detected in untransfected fibroblasts from patients with ABGM2 (Figure 3; Lane 1). However, protein expression was evident in the patient fibroblasts that were transfected with *ssAAV9-GM2A* and collected at both 24- and 48 h post-transfection (Figure 3; compare Lanes 2 and 3). The cells that were collected 48-h post-transfection had almost 9-fold higher GM2A protein expression compared to the cells that were collected 24 h post-transfection. Remarkably, the mice treated with *ssAAV9-GM2A* had almost 2- and 16-fold higher GM2A protein expression than the patient fibroblasts that were transfected with a *GM2A* plasmid and collected at 24- and 48-h, respectively (Figure 3; compare Lane 4 with Lanes 2 and 3, respectively). These data confirm GM2A protein expression in *Gm2a*^−/−^ mice 14-weeks post-injection of *ssAAV9-GM2A*.

### 2.3. ssAAV9-GM2A Reduces GM2 Accumulation in Gm2a^−/−^ Mice

**Figure 4:**
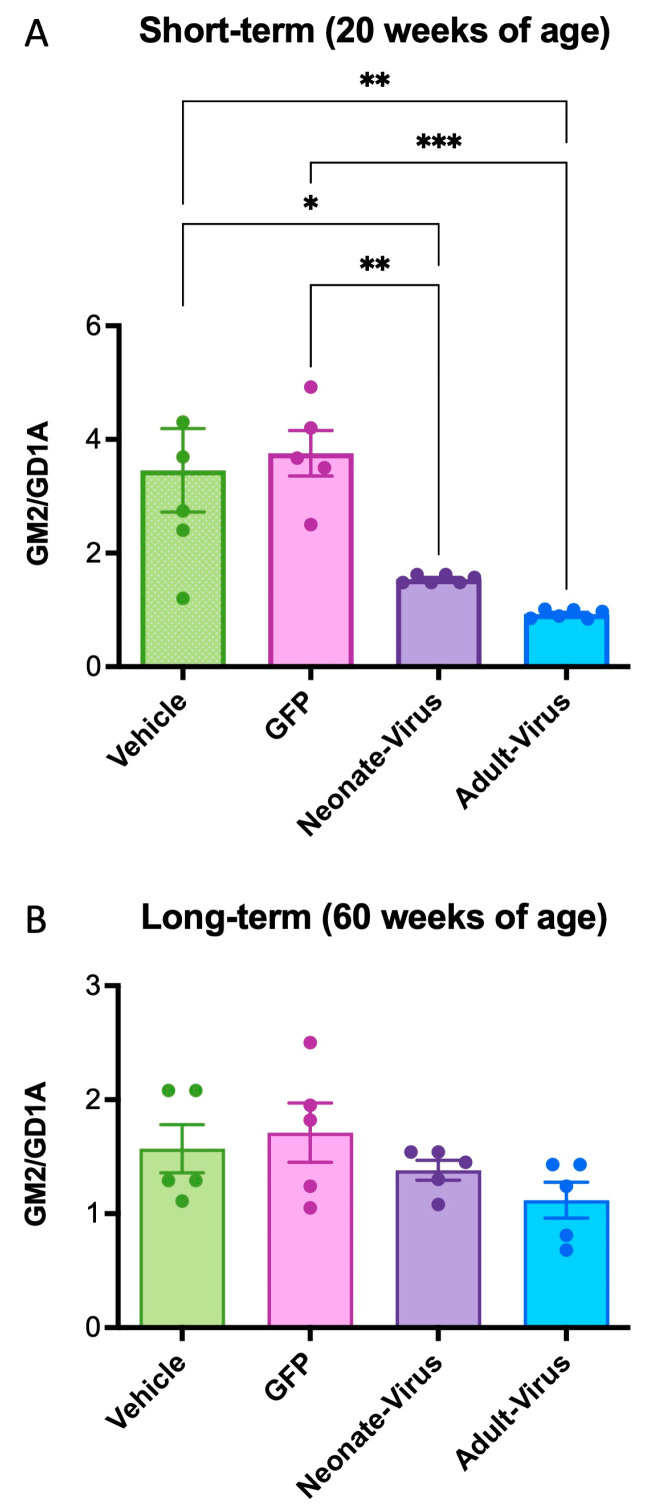
*ssAAV9*-*GM2A* decreased GM2 storage in the mid-section of the brain of *Gm2a*^−/−^ mice at 20 weeks of age and 60 weeks of age. GM2 levels are expressed as a function of GD1a, an internal control that is highly expressed in brain tissue. (**A**) *ssAAV9*-*GM2A* reduced GM2 accumulation in *Gm2a*^−/−^ mice, regardless of age of injection (neonate versus adult) at 20 weeks of age. *Gm2a*^−/−^ mice that were treated at 1-day or 6-weeks of age exhibited significantly less GM2 in their brain than the age-matched *Gm2a*^−/−^ mice injected with PBS (*p* < 0.0189 [*] and *p* < 0.0019 [**], respectively; *n* = 5 or 6/cohort; 1-way ANOVA) or GFP (*p* < 0.0091 [**] and *p* < 0.0010 [***], respectively; *n* = 6/cohort; 1-way ANOVA). (**B**) At 60 weeks of age, mice treated with *ssAAV9-GM2A* neonatally or as adults have reduced GM2 storage compared to both PBS (vehicle) and GFP-injected *Gm2a*^−/−^ mice. However, this difference is not significant. GM2 accumulation is indetectable in heterozygous controls and *is*, thus, not shown on this graph (*n* = 6/cohort).

The short-term (20 weeks of age) and long-term (60 weeks of age) cohorts both showed a reduction in GM2 accumulation in the *Gm2a*^−/−^ mice treated with *ssAAV9-GM2A* compared to the vehicle-treated mice (Figure 4). At 20 weeks of age, untreated mice (*Gm2a*^−/−^ mice injected with PBS or GFP) had 2- to 4-fold more GM2 in their brains compared to neonate- and adult-injected mice, respectively (Figure 4A). Notably, the short-term adult-treated *Gm2a*^−/−^ mice had almost twofold less GM2 accumulation than the neonatally-treated *Gm2a*^−/−^ mice (Figure 4A).

In the long-term cohorts (60 weeks of age), there was no significant effect of treatment in the murine brains, regardless of treatment or age of administration (Figure 4B; compare vehicle-treated *Gm2a*^−/−^ mice with neonate- and adult-virus). However, we were able to deduce a minor decrease in GM2 accumulation in the neonatal- and adult-treated *Gm2a*^−/−^ mice compared to the vehicle-treated *Gm2a*^−/−^ mice—a 12% and a 29% decrease, respectively (Figure 4B).

**Figure 5:**
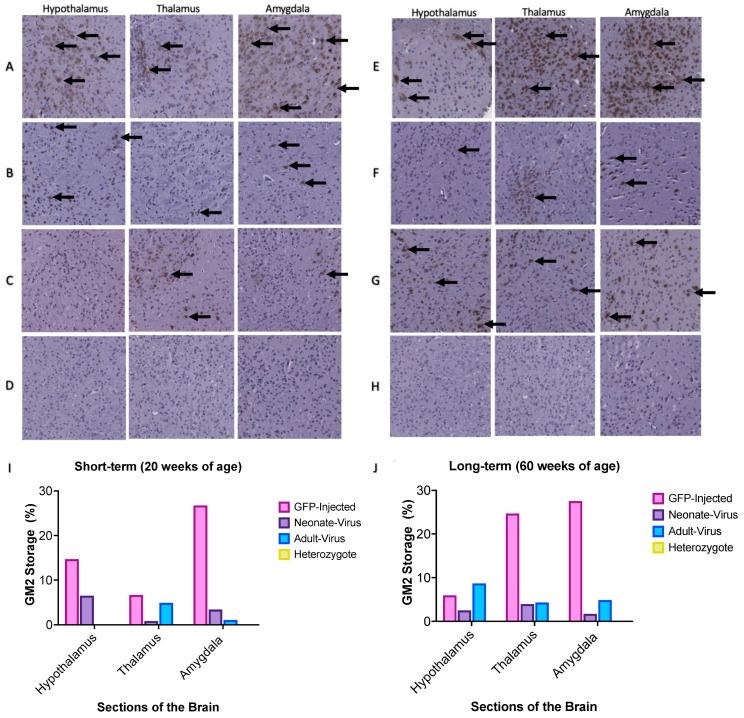
Sections of the murine hypothalamus, thalamus, and amygdala were stained with an anti-GM2 antibody at 15× magnification. The brown spots on the images depict GM2 accumulation. A–C depicts the short-term, 20-week cohort: (**A**) GFP-injected *Gm2a*^−/−^ mice; (**B**) *ssAAV9-GM2A* neonatally-injected *Gm2a*^−/−^ mice; (**C**) *ssAAV9-GM2A* adult-injected *Gm2a*^−/−^ mice; and (**D**,**H**) images from the same pool from disease-free heterozygous mice (*Gm2a*^+/−^) at 60 weeks. E–G depicts the long-term, 60-week cohort: (**E**) GFP-injected *Gm2a*^−/−^ mice (*n* = 1), (**F**) *ssAAV9-GM2A* neonatally-injected *Gm2a*^−/−^ mice, (**G**) *ssAAV9-GM2A* adult-injected *Gm2a*^−/−^ mice. (**I**) In all sections of the brain at 20 weeks, *Gm2a*^−/−^ mice treated with *ssAAV9-GM2A* have less accumulation than GFP-injected *Gm2a*^−/−^ mice. Neonatally injected *Gm2a*^−/−^ mice have much more accumulation in the hypothalamus than adult-treated *Gm2a*^−/−^ mice. However, in the thalamus and amygdala, the adult-injected *Gm2a*^−/−^ mice have notably more accumulation than the neonatally-injected *Gm2a*^−/−^ mice. (**J**) In the thalamus and amygdala, *ssAAV9-GM2A*-treated mice have reduced accumulation compared to GFP-injected *Gm2a*^−/−^ mice. However, in the hypothalamus, *ssAAV9-GM2A* adult-injected *Gm2a*^−/−^ mice have substantially more GM2 than both neonatally-treated *Gm2a*^−/−^ mice and GFP-injected *Gm2a*^−/−^ mice. The black arrow indicates examples of areas saturated with GM2 accumulation.

Histological analysis of the mouse brains was conducted for both short-term (20 weeks of age) and long-term (60 weeks of age) cohorts. Paraffin-embedded brain samples were sliced using a microtome and then stained with an anti-GM2 ganglioside antibody (Figure 5). The GFP-treated *Gm2a*^−/−^ cohorts showed an overall increase in GM2 staining in the hypothalamus, thalamus, and hippocampus, compared to all treated *Gm2a*^−/−^ mice and disease-free heterozygous controls (*Gm2a*^+/−^) at both 20- (Figure 5A–D,I) and 60-week cohorts (Figure 5E–H,J).

### 2.4. There Were No Observable Differences in Behavioural Phenotype between Heterozygous Controls and Gm2a^−/−^ Treated and Untreated Mice

**Figure 6:**
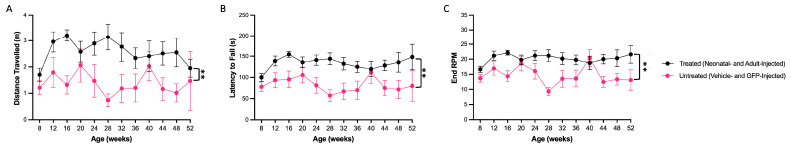
Motor function as assessed by RR tests is improved by *ssAAV9*-*GM2A* treatment between 8 and 52 weeks of age. To evaluate coordination and balance, testing on a rotating rod (RR) was conducted on *ssAAV9*-*GM2A*- or untreated (vehicle- and GFP-injected) cohorts (5 cohorts; *n* = 4–6/cohort, except vehicle-treated cohorts, which only had 2 out of 4 mice survive until 52 weeks of age). The following three parameters were assessed: (**A**) distance traveled, (**B**) latency to fall, and (**C**) end rotations per minute (RPM). Significant improvements were observed in the mice injected with *ssAAV9*-*GM2A* treatment compared to the mice injected with vehicle or GFP in all three parameters (*p* < 0.0053 [**], distance traveled; *p* < 0.0053 [**], latency to fall; *p* < 0.0050 [**], end RPM; *n* = 2 vehicle-injected, *n* = 5 adult-treated; 2-way ANOVA). Individual cohort patterns can be seen in Supplementary Figure S1.

To determine whether correction of GM2 accumulation by *ssAAV9-GM2A* may therapeutically correct motor behavior, RR and OFT tests were conducted on untreated (PBS and GFP) and *scAAV9.hGM2A*-treated cohorts for the 60-week cohorts. The 20-week cohorts were not assessed, as the behavioral phenotypes are relatively mild up to 20 weeks of age [44]. The mice euthanized at 60 weeks of age were assessed for behavioral differences up to 52 weeks of age. There were no differences in motor function as assessed by the OFT (resting time, distance traveled, and mean speed) detected between vehicle- or treatment-treated *Gm2a*^−/−^ mice and the disease-free age-matched litter mates (*Gm2a^+/−^*) (Figure S1). However, over their 60-week lifespan, the *Gm2a*^−/−^ mice that were treated with *ssAAV9-GM2A* at 6-weeks of age performed significantly better than the untreated *Gm2a*^−/−^ mice in all three parameters tested on the RR (distance traveled, end rotations per minute (RPM), and latency to fall) (Figure 6A–C). Although not significant, the *Gm2a*^−/−^ mice that were treated with *ssAAV9-GM2A* at 1-day of age also appeared to perform better than the untreated *Gm2a*^−/−^ mice and the disease-free *Gm2a^+/−^* mice (Figure 6A–C). Notably, there were no significant differences observed in behavioral phenotype between the untreated *Gm2a*^−/−^ mice and the disease-free *Gm2a^+/−^* mice, suggesting that the GM2 accumulation in *Gm2a*^−/−^ mice is insufficient to elicit pathological alterations in motor behavior. Additionally, these findings present preliminary evidence that *ssAAV9-GM2A* may have some degree of neurobehavioural rescue (compare untreated *Gm2a*^−/−^ mice to 1-day and 6-week treated *Gm2a*^−/−^ mice; Figure 6A–C).

## 3. Discussion

This study provides proof-of-concept for *ssAAV9-GM2A* to have therapeutic potential as an intravenous gene therapy for ABGM2. A single transfection (in vitro) of the *ssAAV9*-*GM2A* plasmid into fibroblasts of a patient with ABGM2 or a one-time intravenous administration (in vivo study) of the *ssAAV9*-*GM2A* viral vector (Figure 1) to neonates (1-day of age) or murine early adults (6-weeks of age) was performed. Vector copies were detectable in all examined regions of the brain and the liver in the cohorts that were euthanized at 20 weeks (short-term) and 60 weeks (long-term) of age (Figure 2). The Western blot results confirmed the discernable expression of GM2A protein both in vitro and in vivo (Figure 3), validating the vector design utilized in this study (Figure 1). There was a clear reduction in GM2 accumulation in the brain sections of treated *Gm2a*^−/−^ mice as compared to controls at the 20-week endpoint, but a higher dose may be required to see the same effect in the long term (60-week endpoint) (Figure 4). Copies of the *GM2A* transgene were significantly increased in the livers of *Gm2a*^−/−^ mice that were treated at 6-weeks of age with *ssAAV9-GM2A*; however, this phenomenon was not observed in the mice treated at 1-day of age (Figure 5A). The liver is a well-known sink for systemic AAV infection [34,47,48,49]; thus, these results were not surprising. Liver copy numbers diminished over time, as seen in the 60-week cohort, which is likely due to cell turnover in the liver but not the brain (Figure 5B). At the 60-week endpoint, vector copies in the brain were substantially higher in the *Gm2a*^−/−^ mice treated at 6-weeks of age than they were at the 20-week endpoint; however, this was not observed in the 1-day-old treated *Gm2a*^−/−^ mice (Figure 5B). It is unclear why this was the case, but injection efficiency among different cohorts seems the most plausible explanation. Most importantly, these data demonstrated that the vector therapy was able to cross the BBB and that vector copies were detectable throughout the caudal-, rostral-, and mid-sections of the brain. Thus, a single intravenous injection of *ssAAV9*-*GM2A* at 1.0 × 10^14^ vg/kg per mouse appears to be sufficient for infecting cells of the CNS and producing stable transgene presence up to 1-year post-injection (60-week cohort) at the end of the study. Additionally, the proposed treatment, harboring a human *GM2A* transgene, was administered to a *Gm2a*^−/−^ murine model, thus further confirming that the human GM2A protein can interact with murine β-HexA for catabolism of GM2 in mice [50], as determined by the biochemical GM2 accumulation analysis.

ABGM2 is a progressive neurodegenerative disease. In ABGM2 mice, GM2 begins to accumulate in the brain and spinal cord prior to the onset of symptoms, around 20 weeks of age [44]. Thus, we injected *ssAAV9*-*GM2A* into mice at two different time points: at the neonatal stage (1-day old) and an early adult stage (6-weeks of age) to determine if administration prior to disease onset would be optimal. For metabolic disorders, there are obvious advantages to administering this treatment earlier in life before severe and irreversible brain damage occurs [51]. A recent study compared intravenous administration of gene therapy at multiple disease stages to determine the latest possible point of intervention to observe significant clinical outcomes [52]. Mice with mucopolysaccharidosis, another LSD, were injected with an AAV-mediated treatment at various ages. These data suggest that, regardless of the age of administration, as long as intervention takes place prior to significant neuropathology, it will be efficacious in reversing the accumulation of lysosomal substrates [52]. This is not consistent with the present study, as earlier administration (1-day versus 6-weeks old) did not necessarily result in significantly ameliorated outcomes in terms of disease pathology (GM2 accumulation; Figure 3 and Figure 4).

Our primary outcome measure was to see a reduction of GM2 storage biochemically within the treated mouse brain in both the short-term (20 weeks of age) and long-term cohorts (60 weeks of age) (Figure 3 and Figure 4). There is clear, though partial, rescue of GM2 accumulation in *Gm2a*^−/−^ mice treated with *ssAAV9-GM2A* compared to untreated *Gm2a*^−/−^ mice at both endpoints; a future study may look at higher doses to assess full efficacy potential. Although the differences observed in the 60-week cohort are minimal and not statistically significant (Figure 3B). However, the ability of *ssAAV9-GM2A* to reduce GM2 in *Gm2a*^−/−^ mice suggests that this biochemical correction led to long-term behavioral benefits, especially in the cohort treated at 6-weeks of age (Figure 5). Corrected neurobehavioral outcomes following intravenous administration of AAV-mediated gene therapy are also observed in other mouse models of GM2 gangliosidosis [29,30,31,48]. Additionally, the behavioral data presented here (Figure 5) demonstrates a supranormal amelioration of outcomes in *Gm2a*^−/−^ mice treated with *ssAAV9*-*GM2A* compared to disease-free *Gm2a^+/−^* mice. Although there are observable differences in motor performance when comparing the disease-free *Gm2a^+/−^* mice to the untreated *Gm2a*^−/−^ mice (as seen in the RR assessment), these differences are not significant. This is because of the mild disease manifestation that is observed in *Gm2a*^−/−^ mice [53] due to an alternate metabolic bypass of GM2 degradation in the absence of B-HEXA/GM2A-mediated metabolism presents in mice (and not humans) [54]. In mouse models of GM2 gangliosidosis, GM2 can also be catabolized by sialidases (Neu3) into GA2, which can then be broken down by β-HexA and β-HexB [54]. This pathway is not recognized in humans and can likely explain why *Gm2a*^−/−^ mice do not parallel the disease severity in patients with infantile-onset ABGM2. This is the most notable limitation of our study. Generation of a double knockout mouse by ablating the *Gm2a* and *Neu3* genes to block the main and alternate pathways for GM2 degradation may produce a phenotype closer to the infantile- or juvenile-onset forms of human ABGM2. Future studies are warranted using our approach in a double knockout mouse model (*Gm2a*^−/−^*Neu3*^−/−^) to produce results that may be considered more relevant for translation into a human clinical trial. In addition, testing other variables, such as using an improved vector design employing a self-complimentary AAV9 vector, using a CNS-targeted route of administration, and determining the optimal therapeutic dose, would yield more informative results.

Lastly, though the aim of this study was to generate proof-of-concept, it is imperative to acknowledge the lack of immunological assays reported in this study. Previous studies have demonstrated that an anti-AAV9 capsid immune response is generated in response to AAV9-mediated gene therapy and that a similar anti-transgene response could occur [54,55,56,57], which hinders the long-term benefits of gene therapy treatments. Administering the gene therapy in combination with immunosuppressants could induce immune tolerance [58], thus resulting in more significant outcomes.

This is the first study to show the effects of systemically introducing *ssAAV9*-*GM2A* into an ABGM2 mouse model. The goal of this proof-of-concept study was to use the least invasive method to assess the therapeutic value of introducing an AAV vector containing a human *GM2A* transgene into an ABGM2 murine model (*Gm2a*^−/−^) and its efficacy. We successfully achieved our primary goal of showing a reduction in GM2 accumulation and long-term vector presence. Though further studies are required for optimization, the outcomes of our study provide an initial step towards developing a potential therapeutic strategy for ABGM2.

## 4. Materials and Methods

### 4.1. Plasmid Construct

The vector design for the ssAAV9 vector expressing GM2A is presented in Figure 1. Gene synthesis for the *GM2A* gene was done by BioBasic Inc. (Markham, ON, Canada). The CAG promoter was synthesized by fusing the CMV enhancer with a chicken β-actin (CBA) transcription start site and a rabbit β-globin intron [59]. The viral vector was produced at Dr. Steven Gray’s lab at the University of North Carolina (Chapel Hill, NC, USA).

### 4.2. Western Blot

Fibroblast cells from a donor patient with ABGM2 were kindly donated by Dr. Pesheztski’s lab at McGill University [60] and transfected with *ssAAV9*-*GM2A* using Lipofectamine 2000 (Thermo Fisher Scientific, Mississauga, ON, Canada). Untransfected fibroblast cells were used as a negative control. Liver tissue from a *Gm2a*^−/−^ mouse that was treated with *ssAAV9*-*GM2A* was also extracted. Samples were quantified using the Pierce TM Bicinchoninic Acid (BCA) Protein Assay Kit (Thermo Fisher Scientific, Mississauga, ON, Canada). Proteins were resolved on a 12% sodium dodecyl sulfate polyacrylamide (SDS-PAGE) gel electrophoresis and transferred onto a polyvinylidene fluoride (PVDF) membrane. The membrane was blocked with 5% milk and tris-buffered saline (TBS) and then incubated with the primary anti-GM2A antibody (at that time it was from the Japan group) overnight. Next, the membrane was incubated with a secondary goat anti-mouse antibody (Sigma Aldrich, Oakville, ON, Canada) overnight. The membrane was washed with TBS-Tween (TBST) and imaged using a chemiluminescent reagent (Millipore Sigma, Burlington, MA, USA) on X-ray film. The same steps were conducted to look at β-actin expression on this membrane, with the primary and secondary antibodies being β-actin monoclonal antibody (Sigma Aldrich, Oakville, ON, Canada) and goat anti-mouse antibody (Sigma Aldrich, ON, Canada), respectively.

### 4.3. Animal Models

These studies were performed in mice homozygous for the *Gm2a^tm1Rlp^* targeted mutation, which were purchased from The Jackson Laboratory (Bar Harbor, ME, USA) and have been previously characterized [44]. The Queen’s University Animal Care Committee (Kingston, ON, Canada) approved all the procedures and protocols surrounding the animals.

### 4.4. Injections

A cohort of 1-day-old pups received viral vector in a dose of 1 × 10^14^ vg/kilogram/mouse intravenously, in a total volume of 50 μL, via the superficial temporal vein. Two subsequent controls received equal concentrations and volumes of either green fluorescent protein (GFP) treatment or 1x phosphate buffered saline (PBS). A viral vector in a dose of 1 × 10^14^ vg/kilogram/mouse was also injected intravenously (50 μL) into a cohort of 6-week-old adult mice via tail vein. Mice were monitored and sacrificed in both short-term (20 weeks) and long-term (60 weeks) groups within each cohort.

### 4.5. Behavioral Testing

The experimental mice underwent behavioral tests, including the Rotarod Test (RR) and the Open Field Test (OFT). The testing began at 8 weeks of age and continued monthly until the predetermined endpoint of either 20 weeks or 60 weeks. 

Rotarod

Motor coordination of the mice was assessed using a RR apparatus (IITC Life Sciences, Woodland Hills, CA, USA) as per a previously published protocol [61]. Briefly, mice were placed on moving cylinders that accelerated from 4 to 40 rotations per minute (rpm) over 5 min. Each mouse was given three trials on the RR, with a minimum of 10 min of rest in between. The highest number achieved was reported for each of the following parameters: latency to fall, end rotations per minute, and distance traveled.

Open Field Test

Mice are tested using the OFT (ActiMot, TSE Systems, Berlin, Germany) to assess motor skills [61]. Animals were placed into a square box with high walls for 5 min each to record several parameters. In this study, we analyzed and compared the time spent resting and the mean speed without resting.

### 4.6. Tissue and Serum Collection

Monthly saphenous vein blood collections were done on the experimental mice starting at 8 weeks of age, usually occurring sequentially after behavioral testing. The final blood collection was done at the mouse’s euthanization time point via cardiac puncture. Mice were sacrificed through CO_2_ asphyxiation, followed by cardiac puncture to collect blood and organ perfusion using 1xPBS. Heart, lungs, liver, spleen, kidney, gonads, muscle, brain, and spinal cord were collected for further biochemical and molecular analysis and stored at −20 °C. Tissues collected for histological analysis were submerged in a 4% paraformaldehyde solution and stored at 4 °C.

### 4.7. Ganglioside Storage and Thin Layer Chromatography

An assay to quantify GM2 ganglioside storage was adapted from previous literature [62,63,64]. In short, gangliosides were extracted from sonicated mid-section brain samples using methanol and chloroform solvents. Next, the gangliosides were developed on a thin-layer chromatography plate in a tank containing chloroform, methanol, and calcium chloride (55:45:10). Constituents of the ganglioside mixture travel at different speeds, causing them to separate, allowing us to identify the groups of individual gangliosides. Densitometry analysis (quantification of gangliosides) was performed by comparing the intensity of the GM2 ganglioside bands with the total gangliosides using IMAGE J software Version 1.53. Commercially available ganglioside mix (TLC Monosialoganglioside Mixture; MJS Biolynx Inc., Brockville, ON, Canada) and untreated *Gm2a*^−/−^ mice were used as controls.

### 4.8. Vector Biodistribution

A quantitative polymerase chain reaction (qPCR) method was used to determine the copy numbers of the *GM2A* transgene and *LaminB2* (as controls). GeneAid’s gSYNC^TM^ DNA Extraction Kit (FroggaBio Inc., Concord, ON, Canada) was used to extract the total DNA from each organ, following the manufacturer’s protocol. PowerUp SYBR Green Master Mix (Thermo Fisher Scientific, Mississauga, ON, Canada) on the Applied Biosystems^®^ 7500 Real-Time PCR Systems (Thermo Fisher Scientific, Mississauga, ON, Canada) was used by following the manufacturer’s instructions for each of the reactions. Plasmid DNA was used as the standard for virus quantitation. Purified and quantified mouse genomic DNA was used as a standard for mouse genomic DNA quantitation. Primers for the transgene are as follows:

*GM2A* forward 5′-TATGGGCTTCCTTGCCACTG-3′

*GM2A* reverse 5′-CTCAGGACGCTCTCTATGCG-3′

Mouse LaminB2 primers used for quantification of mouse genomic DNA are as follows:

*LaminB2* forward 5′-GGACCCAAGGACTACCTCAAGGG-3′

*LaminB2* reverse 5′-AGGGCACCTCCATCTCGGAAAC-3′.

Data is shown as the number of viral genomes (vector DNA copies) per mouse genome (vg/mouse).

### 4.9. Histology

Fresh organ tissues were fixed in 4% paraformaldehyde for 24 h, then immersed in 100% ethanol for 24 h before being sent for processing and embedding in paraffin. Paraffin-embedded samples were then sectioned to a thickness of 4–6 μM using a microtome, dried, and baked at 60 °C for overnight. Samples were then deparaffinized, rehydrated, and placed into an antigen retrieval solution. There was blocking of endogenous peroxidase activity as well as non-specific binding. The sections were placed in primary anti-GM2 antibody (KM966, a gift from Kyowa Hakko Kirin Co., Tokyo, Japan) at 1:1000 and then detected using biotinylated human secondary antibody at 1:1000. DAB (3,3′-diaminobenzidine) staining was done, and after this application, the slides were dehydrated initially in 70% ethanol, then 85% ethanol, and lastly in 100% ethanol.

### 4.10. Statistics

Statistical analysis was done using GraphPad Prism 8. The GM2 ganglioside storage assay, biodistribution, and histological analyses were done using one-way ANOVAs with Tukey’s post hoc test. The behavioral testing was analyzed using a two-way ANOVA (mixed effects model) with Dunnett’s multiple comparisons.

## 5. Conclusions

The goal of this proof-of-concept study was to assess the therapeutic potential of *ssAAV9-GM2A* in an ABGM2 mouse model. These data clearly show that *ssAAV9-GM2A* persisted 52-weeks post-injection and that treated animals had reduced accumulation of GM2 in the CNS, which likely led to the observed corrected coordination in *Gm2a*^−/−^ mice. This demonstrates that the proposed vector can efficiently deliver the human *GM2A* transgene to the CNS following systemic administration at both neonatal and early-adulthood stages in mice. These preliminary data pave the way for future optimization studies for gene therapies for ABGM2.

## Data Availability

Data available upon request.

## Supplementary Materials

The supporting information can be downloaded at: https://www.mdpi.com/article/10.3390/ijms241914611/s1.
