# Correction: Vyas et al. Efficacy of Adeno-Associated Virus Serotype 9-Mediated Gene Therapy for AB-Variant GM2 Gangliosidosis. *Int. J. Mol. Sci.* 2023, *24*, 14611

**DOI:** 10.3390/ijms27073166

**Published:** 2026-03-31

**Authors:** Meera Vyas, Natalie M. Deschenes, Karlaina J. L. Osmon, Zhilin Chen, Imtiaz Ahmad, Shalini Kot, Patrick Thompson, Chris Richmond, Steven J. Gray, Jagdeep S. Walia

**Affiliations:** 1 Centre for Neuroscience Studies, Queen’s University, Kingston, ON K7L 3N6, Canada; meera.vyas@queensu.ca (M.V.); 13ko6@queensu.ca (K.J.L.O.); imtiaz_dr@hotmail.com (I.A.); 2 Department of Biomedical and Molecular Sciences, Queen’s University, Kingston, ON K7L 3N6, Canada; zc@queensu.ca (Z.C.); 11sk82@queensu.ca (S.K.); c.richmond@queensu.ca (C.R.); 3 Department of Pediatrics, Queen’s University, Kingston, ON K7L 2V7, Canada; pt24@queensu.ca; 4 Department of Pediatrics, UT Southwestern Medical Center, Dallas, TX 75390, USA; steven.gray@utsouthwestern.edu

In the original publication [1], there was a mistake in the legend for Figure 5. The mistake was the labelling of D and H separately in Figure 5. The slides are from the same pool of mice at 60 weeks of age, as they are better controls for any possible GM2 accumulation to be shown. The correct legend appears below. The authors state that the scientific conclusions are unaffected. This correction was approved by the Academic Editor. The original publication has also been updated.

**Figure 5.** Sections of the murine hypothalamus, thalamus, and amygdala were stained with an anti-GM2 antibody at 15× magnification. The brown spots on the images depict GM2 accumulation. A–C depicts the short-term, 20-week cohort: (**A**) GFP-injected *Gm2a*^−/−^ mice; (**B**) *ssAAV9-GM2A* neonatally-injected *Gm2a*^−/−^ mice; (**C**) *ssAAV9-GM2A* adult-injected *Gm2a*^−/−^ mice; and (**D**,**H**) images from the same pool from disease-free heterozygous mice (*Gm2a*^+/−^) at 60 weeks. E–G depicts the long-term, 60-week cohort: (**E**) GFP-injected *Gm2a*^−/−^ mice (*n* = 1), (**F**) *ssAAV9-GM2A* neonatally-injected *Gm2a*^−/−^ mice, (**G**) *ssAAV9-GM2A* adult-injected *Gm2a*^−/−^ mice. (**I**) In all sections of the brain at 20 weeks, *Gm2a*^−/−^ mice treated with *ssAAV9-GM2A* have less accumulation than GFP-injected *Gm2a*^−/−^ mice. Neonatally injected *Gm2a*^−/−^ mice have much more accumulation in the hypothalamus than adult-treated *Gm2a*^−/−^ mice. However, in the thalamus and amygdala, the adult-injected *Gm2a*^−/−^ mice have notably more accumulation than the neonatally-injected *Gm2a*^−/−^ mice. (**J**) In the thalamus and amygdala, *ssAAV9-GM2A*-treated mice have reduced accumulation compared to GFP-injected *Gm2a*^−/−^ mice. However, in the hypothalamus, *ssAAV9-GM2A* adult-injected *Gm2a*^−/−^ mice have substantially more GM2 than both neonatally-treated *Gm2a*^−/−^ mice and GFP-injected *Gm2a*^−/−^ mice. The black arrow indicates examples of areas saturated with GM2 accumulation. 
