# Supplementary material for: Efficacy of Adeno-Associated Virus Serotype 9-Mediated Gene Therapy for AB-Variant GM2 Gangliosidosis
Source: Int J Mol Sci. 2023 Sep 27;24(19):14611. doi: 10.3390/ijms241914611 (PMC10572999; doi:10.3390/ijms241914611)
Supplement: Supplementary file 1 [file ijms-24-14611-s001.zip › ijms-2400283-supplementary.pdf]

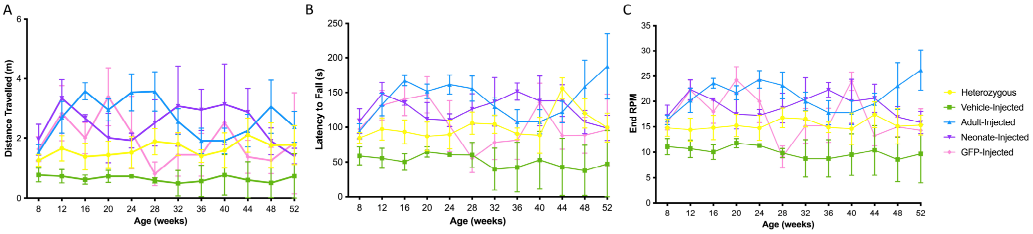

Supplementary Figure S1. Motor function as assessed by RR tests is improved by *ssAAV9-GM2A* treatment between 8 and 52 weeks of age. To evaluate coordination and balance, testing on a rotating rod (RR) was conducted on *ssAAV9-GM2A*- or vehicle-treated cohorts (5 cohorts; n = 4–6/cohort, except vehicle-treated cohorts, which only had 2 out of 4 mice survive until 52 weeks of age). The following three parameters were assessed: (**A**) distance traveled, (**B**) latency to fall, and (**C**) end rotations per minute (RPM).
